# A Comparative Review of Toll-Like Receptor 4 Expression and Functionality in Different Animal Species

**DOI:** 10.3389/fimmu.2014.00316

**Published:** 2014-07-10

**Authors:** Céline Vaure, Yuanqing Liu

**Affiliations:** ^1^Research Department, Sanofi Pasteur, Marcy L’Etoile, France

**Keywords:** toll-like receptor 4, human, non-human primate, mouse, rat, rabbit, swine, dog

## Abstract

Toll-like receptors (TLRs) belong to the pattern recognition receptor (PRR) family, a key component of the innate immune system. TLRs detect invading pathogens and initiate an immediate immune response to them, followed by a long-lasting adaptive immune response. Activation of TLRs leads to the synthesis of pro-inflammatory cytokines and chemokines and the expression of co-stimulatory molecules. TLR4 specifically recognizes bacterial lipopolysaccharide, along with several other components of pathogens and endogenous molecules produced during abnormal situations, such as tissue damage. Evolution across species can lead to substantial diversity in the TLR4’s affinity and specificity to its ligands, the TLR4 gene and cellular expression patterns and tissue distribution. Consequently, TLR4 functions vary across different species. In recent years, the use of synthetic TLR agonists as adjuvants has emerged as a realistic therapeutic goal, notably for the development of vaccines against poorly immunogenic targets. Given that an adjuvanted vaccine must be assessed in pre-clinical animal models before being tested in humans, the extent to which an animal model represents and predicts the human condition is of particular importance. This review focuses on the current knowledge on the critical points of divergence between human and the mammalian species commonly used in vaccine research and development (non-human primate, mouse, rat, rabbit, swine, and dog), in terms of molecular, cellular, and functional properties of TLR4.

## Introduction

Toll-like receptors (TLRs) are among the most studied of the pattern recognition receptor (PRR) families and TLR4 is one of the most studied TLRs. TLR4 specifically recognizes bacterial lipopolysaccharide (LPS) and its activation mainly leads to the synthesis of pro-inflammatory cytokines and chemokines (1, 2). In recent years, the use of synthetic TLR agonists to preferentially stimulate T-helper 1 (Th1) or Th2 immune responses has emerged as a realistic therapeutic goal (2, 3). For example, the TLR4 agonist monophosphoryl A (MPL), a derivative of LPS, has been approved as an adjuvant for human vaccines against hepatitis B and human papilloma virus (4, 5). Other TLR4 agonists are being developed as adjuvant in clinical trials. For instance, GLA (glucopyranosyl lipid adjuvant), a synthetic lipid A, is currently under test in a formulation of stable emulsion (SE) in candidate vaccines against tuberculosis (6). A phase I trial of GLA/SE adjuvanted H5N1 vaccine is complete (7). Such novel and safe adjuvants may facilitate the development of vaccines against poorly immunogenic targets.

Before being tested in humans, an adjuvanted vaccine must be assessed in pre-clinical animal models for safety and efficacy. Such studies are commonly performed in rodents as well as in other animal species. Thus, the extent to which an animal model represents and predicts the human condition is particularly important. The ability of pathogens to evolve quickly has exerted strong evolutionary pressure on the mammalian immune system to adapt in parallel. Due to the various pathogens faced by humans and mice, for example, many aspects of the innate and adaptive immune systems of these two species are known to differ. Thus, human and murine responses to TLR activation have some similarities but also profound differences (8). These differences may affect the predictive value of mouse models for immunological studies, making extrapolation from mouse data to human difficult to achieve. Nevertheless, despite the divergent TLR sequences among species, the basic biological function and down-stream signaling pathways appear to be considerably conserved (Figure 2) (9, 10). This review focuses on the critical points of divergence between the mammalian species commonly used in vaccine research and development, particularly in terms of TLR4 gene, expression patterns, and functionality in innate immunity.

TLRs, first identified in human in the 1990s, are members of the type-1 transmembrane receptor family and are evolutionarily conserved proteins among vertebrates and invertebrates (11–13). TLRs are characterized by an extracellular leucine-rich repeat (LRR) domain involved in ligand recognition and an intracellular toll/interleukin-1 (IL-1) receptor-like (TIR) domain, the latter of which is a highly conserved protein–protein interaction motif module crucial for signal transduction (1, 2). The expression, ligand recognition, and signaling pathways of TLRs, as well as the immune consequences of their activation have been described at length elsewhere (2, 14, 15).

In 1999, Qureshi et al. identified the TLR4 gene in the LPS chromosomal region as responsible for the defective LPS response in some mouse strains. They also identified independent mutations in the TLR4 genes of two LPS-hypo-responsive mouse strains (C3H/HeJ and C57BL10/ScCr), strongly suggesting that TLR4 is essential for mediating responses to LPS *in vivo* (16, 17).

In addition to the recognition of LPS, a major component of the outer membrane of Gram-negative bacteria, TLR4s from various species (e.g., humans and mice) recognize several other components of pathogens such as mannuronic acid polymers from Gram-negative bacteria (18), teichuronic acid from Gram-positive bacteria (19), and viral components such as the F protein of respiratory syncytial virus (20, 21). In addition to exogenous PAMPs, TLR4 also binds endogenous molecules such as heat shock proteins in the mouse (22, 23), in the rat (24), and in the human (25). Fibronectin type III extra domain A (26, 27) and saturated fatty acids (28, 29) are potentially recognized by human and mouse TLR4, and heme by mouse TLR4 (30). Recently, Choi et al. identified cholesteryl ester hydroperoxides, the active components of minimally modified low-density lipoprotein (mmLDL), as a new class of endogenous mouse TLR4 agonists (31). These findings indicated that molecules produced or circulating during abnormal situations, such as during tissue damage, are able to trigger TLR4-dependent pathways (13).

**Figure 1:**
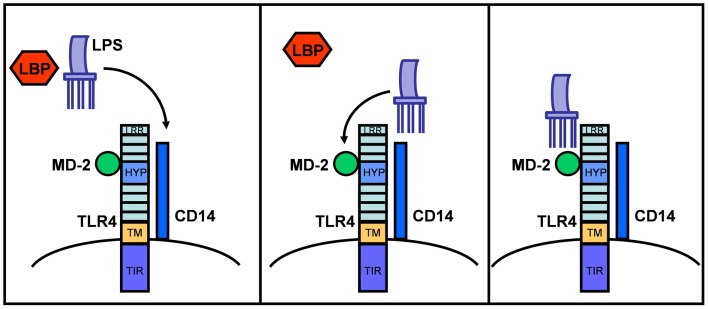
**LPS sensing via LBP and the CD14/MD-2/TLR4 receptor complex**. TLR4 consists of an extracellular domain with leucine-rich repeats (LRR), a hypervariable domain (HYP), a transmembrane domain (TM), and a cytoplasmic domain with a highly conserved TIR-domain. After binding to LBP in serum, LPS is transferred to CD14 and then to the MD-2/TLR4 complex. This illustration is based on mouse and human TLR4 knowledge.

Structurally, TLR4 forms a complex on the cell surface with several other proteins needed for ligand recognition (e.g., LPS) (1, 13, 32, 33). In the serum, LPS is initially bound by LPS binding protein (LBP), which transfers LPS to CD14. CD14 is a glycosylphosphatidylinositol-anchored membrane protein that also exists in a soluble form and that binds LPS–LBP complexes with high affinity. While CD14 itself lacks an intracellular domain for signaling, it associates with TLR4 to form a functional LPS receptor complex. Binding of LPS also requires the MD-2 protein, which associates with the extracellular domain of TLR4 (34). Thus, the active LPS receptor complex includes CD14, TLR4, and MD-2 (Figure 1), although further study suggests that CD14 and LBP only enhance the TLR4-dependent LPS signaling and are not absolutely required for LPS binding and signaling (35).

TLR4 ligands appear to bind the TLR4/MD-2 complex rather than TLR4 alone. It is possible that MD-2 partially determines the binding specificity of the TLR4/MD-2 receptor complex for LPS variants and consequently affects TLR4 function in different animal species (35, 36). Thus, paying special attention to MD-2 activities across species may be informative in investigations of TLR4 function and in identifying relevant animal models.

## TLR4 Signaling

### MyD88-dependent and MyD88-independent signaling pathways

TLR4 signaling pathways have been reviewed extensively by others (14, 15, 32, 37, 38). Briefly, upon ligand binding at the cell surface, TLR4 receptors homodimerize through interactions between their intracellular TIR-domains, resulting in conformational changes in the molecule. The subsequent signaling process involves the recruitment of TIR-domain-containing adapter molecules to the cytoplasmic face of the TLR4 cluster via homophilic interactions between the TIR-domains. Four TIR-domain-containing adapter molecules belonging to two distinct pathways are known to mediate TLR4 signaling: Myeloid differentiation factor 88 (MyD88); MyD88-adapter-like (Mal) protein, also known as TIR-domain-containing adapter protein (TIRAP); TIR-domain-containing adapter inducing interferon-β (TRIF), also called TIR-domain-containing adapter molecule-1 (TICAM-1); TRIF-related adapter molecule (TRAM), also called TIR-containing protein (TIRP), or TIR-containing adapter molecule-2 (TICAM-2). TLR4 requires all four of these adapters to mediate a comprehensive immune response.

**Figure 2:**
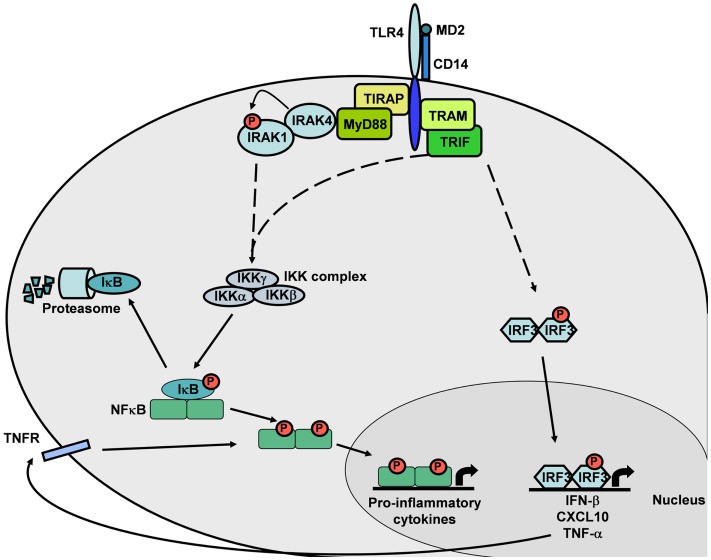
**TLR4 intracellular signaling cascade**. The MyD88-dependent pathway induces NF-κB translocation and expression of pro-inflammatory cytokine genes. The MyD88-independent pathway induces NF-κB or IRF3 translocation, leading to expression of pro-inflammatory cytokine genes by NF-κB or IFN-β and TNF-α genes by IRF3. This canonical signaling pathway is based on published mouse and human TLR4 knowledge.

TLR4 initiates intracellular signaling by at least two major pathways: (i) the TIRAP–MyD88 pathway, which regulates early NF-κB activation and related inflammatory cytokine production, such as IL-12; and (ii) the TRIF–TRAM pathway, which activates the interferon regulatory factor-3 (IRF3) transcription factor that effectuates the subsequent up-regulation of genes encoding type I interferons (IFNs) and co-stimulatory molecules. This TRIF-dependent pathway also activates TNF-α production and secretion. The subsequent binding of secreted TNF-α to its receptors leads to NF-κB activation. Thus, the TRIF–TRAM pathway is also responsible for the late phase NF-κB activation through IRF3 and TNF-α secretion (Figure 2). MyD88-independent signaling accounts for the majority of the LPS response. The MyD88-independent pathway results in the induction of dendritic cell (DC) maturation (consequent to the expression of the genes encoding co-stimulatory molecules such as CD40, CD80, and CD86) and elevated expression of type-1 interferon genes and of IFN-regulated genes (14, 32, 39).

### SARM: TLR4 signaling pathway inhibitor

A fifth TIR-domain-containing adaptor, SARM (sterile alpha and HEAT/Armadillo motif), was characterized (38, 40). Expression of the gene encoding SARM is induced by LPS-mediated activation of TLR4 (41). SARM strongly inhibits TRIF-mediated, but not MyD88-mediated, activation of NF-κB. In resting cells, SARM and TRIF appear to interact weakly with each other. Following TLR4 activation, this interaction stabilizes and the resulting complex prevents TRIF from interacting with other TLR adapter proteins. Consequently, SARM is a negative regulator of TRIF-mediated TLR signaling. This negative modulation may prevent innate immune cells from an excessive activation in response to LPS that may lead to lethal septic shock.

## Molecular, Cellular, and Functional Properties of TLR4 Across Different Mammalian Species

### Similarities and differences in TLR4 gene and amino acid sequences across species

**Figure 3:**
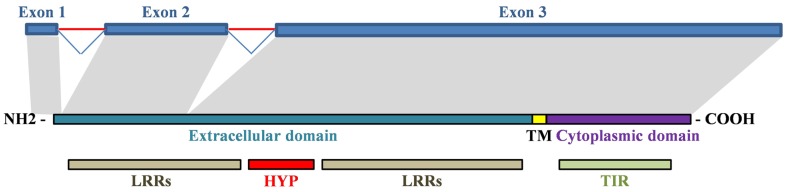
**Alignment of human TLR4 gene and protein**. Exon 1 encodes a signal peptide and initial amino acids of the extracellular domain. Exon 2 encodes first LRRs in the extracellular domain. Exon 3 encodes the remaining extracellular domain (hypervariable region and LRRs), the transmembrane domain and the cytoplasmic domain. TM, transmembrane domain; HYP, hypervariable region.

Adaptive evolution is particularly prevalent in immune response genes as a result of the sustained selective pressure exerted by rapidly evolving pathogens (8). Mutations in the TLR4 gene can inhibit the immune responses against the pathogens specifically recognized by the ancestor receptor. In this regard, gene evolution across species can lead to different TLR4 gene expression patterns or changes in the receptor’s affinity and specificity to its ligands. Consequently, polymorphisms in the coding or promoter sequence of TLR4 in different species may lead to different resistance/susceptibility patterns to infectious diseases (10, 42). Human TLR4 gene is composed of three exons. Their alignment to the TLR4 protein is illustrated in Figure 3.

As indicated in Table 1, TLR4 genes are highly conserved across mammalian species. Among different TLR4 sub-regions, the intracellular TIR-domain is highly conserved across species suggesting that the signal transduction pathways of TLR4 are also similar across species. By contrast, TLR4 extracellular domains exhibit considerable sequence divergence and the LRRs are highly polymorphic (10, 42, 43). Species-specific LRR variants in TLR4 may be due to variations in the ligand-binding process and to the different roles of the TLR4 co-receptor molecules. For example, Lizundia et al. described a species-specific restriction in LPS recognition, potentially due to differences in the binding of MD-2 to the LRRs of the TLR4s of different species (35).

**Table 1:** **Identity (%) between exon 1-cds, exon 2, and exon 3-cds nucleotide sequences of human TLR4 gene and that of various animal species [based on data from Ref. (42)]**.

Animal species	Pig	Mouse	Gorilla	Orangutan	Chimpanzee	Baboon
Exon 1-cds	80	75	93	92	100	87
Exon 2	87	77	99	99	100	97
Exon 3-cds	81	74	99	97	99	94

Pair-wise alignment of the extracellular domain of human TLR4 with those of other species indicates that the most divergent region of TLR4 is its ligand recognition domain, in which different species exhibit a wide variety of surface electric charge (42–44). The first 82-amino acids of the proximal region of the TLR4 extracellular domain are poorly conserved across species and are highly variable for individuals in the same species. This 82-amino acid hypervariable region is known to be involved in species-specific recognition of several TLR4 ligands as discussed later in Section “Expression Pattern and Functionality of TLR4 Across Species – Mouse”.

#### Human

The human TLR4 gene maps to chromosome 9q32–33 (16), SSC9:119.5 (45) and is composed of three exons (42) (Table 2). Its pre-mRNA sequence is 11467 base pairs (bp) long from the 5′ cap to the 3′ end of the transcribed sequence (46). The human TLR4 consists of an extracellular domain of 624 amino acids (residues 1–624), a transmembrane domain of 33 amino acids (residues 625–658), a proximal cytoplasmic domain of 159 amino acids (residues 659–818), and a distal cytoplasmic domain of 19 amino acids (residues 819–838). The ectodomain consists of 21 LRRs (amino acids 55–569 of the extracellular domain) (47). Two TLR4 mRNAs of ~5.5 kb and 4.4 kb in size can be detected in all tissues and are created by alternative splicing of the 3′UTR domain of the pre-mRNA transcript (17). The human TLR4 also displays many single nucleotide polymorphisms (SNPs) particularly in the ectodomain of the protein, although most of these have only mildly deleterious phenotypic effects (48).

**Table 2:** **Comparison of the organization of human, porcine, and murine TLR4 genes**.

Animal species	Human	Pig	Mouse
Exon 1-cds	93^a^	93	93
Intron 1	3996	3866	5972
Exon 2	167^b^	167	167
Intron 2	3638	2582	5168
Exon 3-cds	2260	2265	2251
Exon 3-3′-UTR	1127	568	1313

*Values are in base pairs*.

*^a^Same as the pygmy chimpanzee, and olive baboon gene*.

*^b^Same as the gorilla, orangutan, pygmy chimpanzee, and olive baboon gene. Data from Ref. (42)*.

#### Non-human primate

TLR4 of non-human primates (NHP) bears great similarity to its human counterpart (Table 1) (46). However, some differences exist even among NHPs. For instance, humans and chimpanzees are generally considered to be very sensitive to LPS, whereas baboons are highly resistant (46, 49). The human and chimpanzee TLR4 amino acid sequences share 99–100% identity, irrespective of the domain, distinguished by only three substitutions, except for the 82-amino acid hypervariable regions, which are 98% identical (44, 46). The baboon and human TLR4 amino acid sequences share 91.5% similarity in the extracellular domain and 85% similarity in the 82-amino acid hypervariable region. The baboon TLR4 transmembrane domain sequence differs from the human sequence in one out of 30 residues, and in the proximal cytoplasmic domain by only 1 out of 155 amino acids. The carboxyl terminal domain is less similar to its human counterpart, with 16 of the last 21 human residues not replicated in the baboon protein, resulting in a protein that is 13 amino acids shorter than the human protein (Table 3). The authors suggested that this variation in the distal cytoplasmic region of TLR4 might be a reason for interspecies differences in LPS sensitivity. However, since TIR is instead located in the proximal cytoplasmic region, whether such mutations were functionally essential was in question (46).

**Table 3:** **Sequence similarity (%) between the amino acid sequences of human TLR4 and those of the chimpanzee, baboon, mouse, rat, and rabbit [based on data from Ref. (44, 46, 47)]**.

Animal species	Chimpanzee	Baboon	Mouse	Rat	Rabbit
Distal extracellular domain	100	95	66	66	77
Hypervariable region	98	85	48	39	57
Proximal extracellular domain	100	93	63	64	63
Transmembrane domain	100	97	70	68	75
Proximal cytoplasmic domain	99	99	90	92	
					85
Distal cytoplasmic domain	100	50	26	38	

The amino acid sequences of the NHP TIR-domains have not been altered in the course of evolution. Indeed, most mutations in these TIR-domains would likely have been deleterious. The TIR-domains of the rhesus macaque display a high level of similarity with their respective human counterparts with only two differences out of 169 amino acids. No differences are found between the gorilla and human TIR-domains of TLR4 (49).

#### Mouse

The mouse TLR4 gene maps to chromosome 4, SSC4:66.5 (45) and is made up of three exons (42) (Table 2). The mouse gene produces a longer pre-mRNA sequence than its human counterpart, i.e., 15337 bp versus 11467 bp, respectively (46). Each murine exon corresponds to a homologous sequence in the human gene. A number of conserved promoter and enhancer motifs can be aligned in the murine and human 5′ flanking sequences. Murine and human TLR4 share 67–71 and 79–81% similarity at the nucleotidic and amino acid levels, respectively (17, 35). Amino acid similarity between the mouse and human TLR4 sequences is 62% in the extracellular domain, 70% in the transmembrane domain, and 83% in the cytoplasmic domain (44, 46, 47). The 82-amino acid hypervariable region is the least conserved between the two species with only 48% similarity. In the cytoplasmic region, the amino acid conservation between the mouse and human is greater in the proximal domain (TIR-domain) (90%) than in the distal domain (26%) (Table 3). In the TIR-domain, only 12 out of 169 amino acids differ between the mouse and human TLR4 sequences (49). The human and murine promoter sequences share only 53% similarity and the components involved in the regulation of gene expression display significant differences (1, 42).

The murine and human MD-2 proteins share mere ~57% amino acid similarity (47). Mouse MD-2 also displays some discrete structural differences relative to the human counterpart in terms of electric charge properties (36). Human MD-2 is more cationic than mouse MD-2. This disparity includes residues close to the LPS binding site that are positively charged in the human MD-2. These differences impact not only the secretion but also the function of MD-2, thus the activation of TLR4.

Across mouse strains, the TLR4 locus exhibits some genetic variations too. Among 35 strains of *Mus musculus*, 10 different alleles are identified on the basis of mutations at 22 sites, compared to a common reference sequence (46). Some strains have accumulated more mutations than others. For example, the LPS-tolerant C3H/HeJ strain harbors a single C to A transversion point mutation, which results in a non-conservative amino acid substitution (proline 712 to histidine 712) within the cytoplasmic domain of TLR4 (16, 17, 46). This substitution is expected to alter the topology of the TLR4 signaling domain and potentially disrupt protein–protein interactions with down-stream molecules. Indeed, the P712H substitution in TLR4 results in a co-dominant inhibitory effect on LPS signal transduction. Another endotoxin-tolerant strain is the C57BL/10ScCr mouse, which harbors a genomic deletion in the Lps locus and does not produce a TLR4 transcript or a TLR4 protein. These observations support the hypothesis that their mutant phenotype is due to a loss of TLR4 function.

The C57Bl/6J, DBA2, BALB/c, C3H/ARC, and C3H/HeJ mouse strains differ in at least two genetic loci known to influence innate immune responses (50). The C3H/HeJ strain is known to be LPS hypo-responsive, whereas the other four strains are LPS sensitive. Four hundred fifteen genes are identified by cDNA microarray analysis as LPS-inducible and temporally regulated in bone marrow-derived macrophages of the four LPS-responsive strains. These genes are not regulated by LPS in cells from the C3H/HeJ mice. Thus, this set of genes represents possible down-stream targets specific to TLR4 signaling pathways. The DBA2 and C57Bl/6J mouse macrophages share a similar temporal profile of LPS-inducible gene regulation, but the gene induction is delayed in the BALB/c and C3H/ARC macrophages. Most of the genes induced encode components of the cytoskeleton or the phagosome, which correlates with the morphological changes that occur in bone marrow-derived macrophages upon LPS stimulation. Thirty elements corresponding to 22 genes involved in cell growth, cycling, and differentiation belong to the TLR4-dependent transcriptional pathway.

#### Rat

In the extracellular domain of TLR4, rats and humans share 61% overall amino acid similarity (46). The 82-amino acid hypervariable region is the least conserved with only 39% amino acid similarity, whereas the similarity is ~65% for the remainder of the extracellular domain (44). Amino acid similarity is 68% in the transmembrane domain, 92% in the proximal cytoplasmic region, and 38% in the distal cytoplasmic region (46) (Table 3). Rats and humans display 10 amino acid differences in the TLR4 TIR-domain out of 169 amino acids (49).

#### Rabbit

Comparing to the mouse and rat, the TLR4 and MD-2 proteins from the rabbit are highly similar to their human counterparts, with 72 and 70% amino acid similarity, respectively (47, 51). In the TLR4 extracellular domain, the distal region displays the greatest shared similarity (77%), whereas the 82-amino acid hypervariable region displays the lowest similarity (57%). The cytoplasmic and transmembrane domains of the rabbit TLR4 share 85 and 75% similarity respectively to their human counterparts (Table 3).

Because the amino acid similarity shared with the human 82-amino acid hypervariable region is greater in the rabbit than in the mouse, the rabbit TLR4 may recognize human pathogens better than the mouse TLR4 (47). This, and the greater overall similarity between rabbit and human TLR4, suggests that the human immune response to some pathogens may be better modeled in rabbits than in mice.

#### Swine

The porcine TLR4 gene maps to chromosome 1, SSC1:284.7 (45). As in humans and mice, the porcine TLR4 gene is made up of three exons. The open reading frame (ORF) of 2526 bp encodes 841-amino acid protein (42). The first exon includes a 98 bp 5′-UTR and the first part of the coding sequence; the third exon encodes the last 754 amino acids of the TLR4 protein along with a 568 bp 3′UTR (Table 2). Overall, the predicted porcine TLR4 protein contains a 23-amino acid putative leader peptide, an extracellular domain of 608 residues (24–632), a hydrophobic transmembrane region of 21 residues (633–653), a proximal cytoplasmic region (654–672), and a cytoplasmic TIR-domain of 147 residues (673–819). The extracellular domain includes 21 LRRs of 20 to 29 residues (42, 52).

Porcine and human TLR4 nucleotide sequences share 65-77% similarity (51). The porcine TLR4 amino acid sequence is 63–80% similar to the complete human, rabbit and mouse TLR4 sequences. Again, most of the amino acid differences among these species are located in a region between residues 285 and 366, which corresponds to the 82-amino acid hypervariable region involved in ligand recognition (52). In the cytoplasmic portion, the TIR-domain is the most conserved segment and is over 90% similar to the human, rabbit, and mouse TIR-domains. Porcine and human promoter sequences are 71% similar. Overall, the porcine TLR4 promoter shares more features with the human TLR4 promoter than its murine counterpart (42).

#### Dog

The full-length cDNA sequence of canine TLR4 (2709 bp) contains a 5′ UTR (1–194 bp), a long ORF (195–2105 bp), a 3’ UTR (2106–2687 bp) and encodes a protein of 637 amino acids (53). Canine TLR4 is 70–77% similar to the human TLR4 nucleotide and amino acid sequences, and is highly similar to the sequences of other mammalian TLR4s. However, the canine gene lacks the two first exons found in other TLR4 genes (42, 53). Sequence alignment confirms that this is not due to a loss of introns or a different exon sequence organization (Accession numbers: Genbank O00206 and Genbank BAB85609.1).

### Expression pattern and functionality of TLR4 across species

Despite extensive sequence similarity among mammalian TLR4s, species-specific variations in their extracellular domains cause these receptors to have different spectrums of agonists and antagonists. In addition, there is substantial diversity in the TLR4 cellular expression pattern and tissue distribution. Consequently, TLR4 functions vary across different species (35). Table 4 summarizes the TLR4 expression patterns in the following species: humans, NHP, mice, rats, swine, rabbits, and dogs.

**Table 4:** **TLR4 expression across species**.

Organs	Human	Non-human primate	Mouse	Rat	Swine	Rabbit	Dog
**PBL**	+++	+++	+++	ND	ND	ND	+++
Myeloid subsets	+++	+++	+++	ND	ND	ND	ND
Monocytes	+++	ND	+++	+	+/−	ND	ND
Macrophages	+++	ND	+++	ND	+/−	ND	ND
Granulocytes	+++	ND	+++	ND	ND	ND	ND
Dendritic cells			+++	+/++		ND	ND
Immature dendritic cells	+	+	+	ND	+++	ND	ND
Mature dendritic cells	−	−	ND	ND	+++	ND	ND
Plasmacytoid dendritic cells	−	−	+	+	ND	ND	ND
Lymphoid subsets	+/−	+/−	++	ND	ND	ND	ND
**Adipocytes**	++	ND	ND	ND	ND	ND	ND
**Brain**	+	ND	ND	+	+		ND
Basilar artery	ND	ND	ND	ND	ND	+	ND
Hypothalamus	ND	ND	ND	ND	+	ND	ND
Hippocampus	ND	ND	ND	ND	+	ND	ND
Cortex	ND	ND	ND	ND	+	ND	ND
Cerebellum	ND	ND	ND	ND	+	ND	ND
Microglia	++	ND	++	++	ND	ND	ND
Astrocytes	+	ND	−	+/−	ND	ND	ND
Neurons	ND	ND	ND	+/−	ND	ND	ND
Oligodendrocytes	+	ND	−	−	ND	ND	ND
**Cornea**	ND	ND	ND	+	+	ND	+
**Endothelium**
Dermal microvessel endothelium	++	ND	ND	ND	ND	ND	ND
Umbilical vein endothelium	++	ND	ND	ND	ND	ND	ND
Lung endothelium	ND	ND	ND	ND	ND	++	ND
Brain endothelial cell line	++	ND	ND	ND	ND	ND	ND
Primary brain endothelial cells	ND	ND	ND	++	ND	ND	ND
**Heart**	+	ND	+++	+	ND	+	ND
**Intestine**
Small Intestine	++	ND	ND	ND	+/++	ND	++
Colon	++	++	ND	ND	++	ND	+
Jejunum	ND	ND	ND	ND	+/++	ND	ND
Ileum	++	++	ND	ND	++	ND	++
Duodenum	ND	ND	ND	ND	ND	ND	++
Intestinal epithelial cell lines	++	ND	ND	ND	ND	ND	ND
Mesenteric lymph node	ND	ND	ND	ND	ND	ND	+++
**Kidney**	+	ND	++	ND	+	++	+/−
**Liver**	+	ND	++	ND	+	+	+
Kupffer cells	ND	ND	ND	ND	+	ND	+
Hepatocytes	ND	ND	ND	ND	−	ND	−
Vascular endothelium	ND	ND	ND	ND	−	ND	−
Bile duct epithelium	ND	ND	ND	ND	+	ND	+
**Lung**	++	ND	+++	ND	+	++	+
Macrophages	ND	ND	ND	ND	+	++	+
Epithelial cells	ND	ND	ND	ND	+	−	+
**Lymph node**	ND	ND	ND	ND	++	ND	ND
**Ovary**	++	ND	ND	ND	+	ND	ND
**Pancreas**	+	ND	+	ND	ND	ND	ND
Insulin-producing β-cells	+	ND	+	ND	ND	ND	ND
Glucagon-secreting α-cells	+/−	ND	ND	ND	ND	ND	ND
**Placenta**	++	ND	ND	ND	ND	ND	ND
**Prostate**	+	ND	ND	ND	ND	ND	ND
**Skeletal muscle**	−	ND	ND	ND	−/+	ND	−
**Skin**	−	ND	ND	ND	−	ND	−
**Smooth muscle**	+	ND	++	ND	−	ND	−
**Spleen**	+++	ND	+++	ND	++/+++	++	++
**Stomach**	ND	ND	ND	ND	ND	ND	++
**Testis**	+	ND	ND	ND	ND	ND	ND
**Tonsil**	ND	ND	ND	ND	++/+++	ND	ND
**Thymus**	+	ND	ND	ND	++	+/−	ND

*Summary of TLR4 expression patterns in the human, non-human primate, mouse, rat, swine, rabbit, and dog to our knowledge (see text for references, ND, not described)*.

#### Human

The predominant TLR4 expressing cells in humans are of myeloid origin. Analyses of total RNA showed that TLR4 mRNA was present only in myeloid cells and was undetectable in resting or activated lymphoid cell subsets. Human plasmacytoid dendritic cells (pDCs) do not express TLR4 (9, 54–56). Human B cells were thought to lack significant TLR4 expression, at least in the naïve resting state, and were thus considered to be unresponsive to LPS (9, 57). However, TLR4 expression in human B cells may be inducible by specific stimuli, such as IL-4, suggesting that B cells can be rendered sensitive to TLR4 ligands (58).

Characteristic patterns of TLR4 and MD-2 expression are observed in monocytes, immature DC, and mature DC. Monocytes produce a high level of TLR4 mRNA (54). This TLR4 expression level decreases markedly during immature DC formation but remain clearly detectable (59). Further DC maturation is associated with a loss of TLR4 expression and the consequent loss of LPS responsiveness. The inverted MD-2/TLR4 ratios in monocytes and DCs suggest that MD-2 might limit the LPS response in monocytes whereas TLR4 would limit DC response. DC activation by TLR4 agonists induces up-regulation of the genes encoding IL-12 p70, IP-10, and IFN-β (60). Exposure of monocytes and granulocytes to LPS or to pro-inflammatory cytokines increases the expression of TLR4 (55). Similarly, stimulation of THP-1 (a monocyte-like leukemic cell line) by PMA (phorbol 12-myristate 13-acetate) increases the sensitivity of these cells to LPS that correlates with marked increases in TLR4, CD14, MD-2, and MyD88 mRNA levels (56). In addition to the classical increase of TLR4 expression by PAMPs, TLR4 expression in monocytes may also be regulated by glucose, as high concentrations of glucose but not mannitol induce TLR4 mRNA and protein expression and TLR4 activation (61, 62).

In human tissues, the highest levels of TLR4 mRNA detected by PCR are in the spleen and PBLs. Moderate levels of TLR4 expression are detected in the colon, ovary, lungs, small intestine, and placenta, whereas TLR4 expression is low in the brain, heart, kidneys, liver, prostate, pancreas, testis, muscle, and thymus. No TLR4 expression was detected in skeletal muscle or skin (53, 56, 62). Further investigations using for example immunohistochemistry, could help to determine whether TLR4 is expressed by tissue cells or by cells of blood origin, in particular for organs like liver and lungs. TLR4 is constitutively expressed in the human fat tissue with a clear detection in adipocytes, and its expression is not regulated following LPS treatment (63). Nevertheless, stimulation of mature adipocytes with a TLR4 agonist increases TNF-α protein concentrations. Since mature adipocytes do not express CD14, the activation of TLR4 on these cells likely requires exogenous CD14. In cultured human corneal epithelial cells and corneal stromal fibroblasts, *Acanthamoeba* challenge up-regulated TLR4 expression, and induced an early production of IL-8 and TNF-α through the TLR4/NF-κB pathway and later production of IFN-β through the TLR4/Erk1/2 pathway (64).

In the human central nervous system (CNS), TLR4 is expressed by two types of non-neuronal supportive cells: the CNS residential macrophages or microglia and the macroglial cells such as astrocytes (65). In microglia, TLR4 protein is localized within intracellular vesicles and is undetectable on the cell surface (only ~15% of microglia cells express TLR4). Astrocytes and oligodendrocytes also slightly express TLR4. In astrocytes, TLR4 is exclusively localized on the cell surface and is not detectable within intracellular vesicles. This difference in the TLR4 subcellular localization may be linked to the different phagocytic and antigen processing properties of microglia and astrocytes. Development of multiple sclerosis lesions in humans is associated with higher levels of TLR4 expression. The TLR4 mRNA level increases in a human brain endothelial cell line following oxidative stress (66). TLR4 may be involved in cardiovascular diseases. For example, elevated levels of TLR4 have been found in the tissues of failed hearts and ischemic hearts (67).

Non-diabetic human β-cells contain TLR4 and CD14 transcripts (62). Pancreatic islets subjected to flow cytometric analysis displayed variable levels of TLR4 protein on the cell surfaces, with the TLR4 found almost exclusively on insulin-producing β-cells. TLR4 and CD14 expression increased following LPS exposure in a dose-dependent manner. In the presence of LPS-induced TLR4 expression in β-cells, insulin mRNA, insulin secretion, and cell viability decrease.

Human dermal microvessel endothelial cells (HMEC) and human umbilical vein endothelial cells (HUVEC) express TLR4 (68). However, unlike monocytes, macrophages, and pancreatic β-cells, endothelial cells do not express CD14. Thus, activation of endothelial cells by LPS requires the presence of soluble CD14 in the serum. Expression of TLR4 has been also detected in intestinal epithelial cell lines (69, 70). Although normal human ileal epithelium barely expresses TLR4, its expression is up-regulated in inflammatory bowel disease (IBD). This phenomenon may explain why large quantities of luminal LPS are usually well tolerated by healthy intestine since the low levels of TLR4 minimize LPS recognition. However, this tolerance toward luminal bacterial toxins may be broken during IBD as a result of TLR4 up-regulation.

#### Non-human primate

Although the currently available evidence is limiting, no fundamental differences between humans and NHPs at the level or pattern of TLR4 expression have been reported. Blood DC subsets, DCs derived from monocytes *in vitro* (mo-DCs), monocytes, and B cells of rhesus macaques display the same expression patterns of TLRs (TLR3, -4, -7, -8, and -9) as those of humans (9). Neither macaque nor human pDCs express TLR4. Consequently, these pDCs do not undergo any differentiation or morphological changes in response to stimulation with *E. coli* LPS. LPS exposure of macaque monocytes induces CD40 expression and a high level of TNF-α production, but no CD86 expression. LPS stimulation of macaque myeloid DCs and mo-DCs up-regulates the expression of CD40 and CD86, but induces only low levels of TNF-α production and no detectable IL-12p70. In addition, TNF-α and IL-6 synthesis by macaque myeloid DCs and monocytes following LPS exposure is regulated in an age-dependent manner (71).

For macaque PBMCs, Asquith et al. recently reported that TLR4 and SARM expression levels were lower in juvenile macaques (1–7 years old) than in adult (5–18 years old) or aged animals (≥19 years old) (71). As SARM is a negative regulator of TLR4 signaling by blocking TRIF binding to TLR4 adapter proteins (41, 72), a low level of negative regulation by SARM may compensate the low level of TLR4 expression in young macaques and allow a robust response to LPS.

Immunochemistry studies revealed that TLR4, MD-2, NF-κB, and IRAK-1 are constitutively expressed at low levels in the ileal epithelium of adult macaques (70, 73). Intestinal epithelial cells normally restrict the entry of LPS in the circulation. However, inflammatory or non-inflammatory (e.g., stress) conditions can induce the translocation of LPS into the blood circulation. Given that TLR4 is expressed in the apical membrane of ileal epithelial cells, the presence of LPS in the ileal lumen may stimulate epithelial cells through TLR4 and induce the production of chemoattractant factors for neutrophils and inflammatory cytokines. LPS that has been translocated from the villous epithelium can then be taken up by neutrophils, which then migrate to the portal circulation via the epithelium and lamina propria.

Data are limited regarding the function of TLR4 in NHP species other than macaques. Nevertheless, species-specific similarities and differences have been noted among different NHP species. For example, human and baboon arterial endothelial cells display a low but similar basal expression of TLR4 (74). However, TLR4 expression is up-regulated by LPS only in the human cells. In addition, only the human NF-κB pathway is activated upon LPS exposure despite the fact that the NF-κB machinery is intact in both human and baboon arterial endothelial cells. This could explain why baboon arterial endothelial cells appeared being resistant to the pro-inflammatory effects of LPS and the lack of vascular response to LPS in baboons. This lack of activation of the TLR4 signaling pathway appears to be cell specific since human and baboon PBMCs are similarly responsive to LPS.

#### Mouse

In mice, as in humans, cells of myeloid origin such as monocytes, macrophages, microglia, myeloid DCs, and granulocytes exhibit the highest levels of TLR4 expression. TLR4 is also expressed by lymphoid cell types like naïve B cells and freshly isolated T cells. However, in sharp contrast to human pDCs, murine pDCs express high levels of TLR4 (9, 57, 75).

Minimally modified LDL (mmLDL), via its cholesteryl ester hyperoxide moieties, increases macro-pinocytosis by mouse macrophages in a TLR4-dependent manner (31). This leads to lipid accumulation in macrophages. mmLDL also induces Syk recruitment to TLR4. This process leads to actin polymerization, a step required for membrane ruffling that likely promotes antigen uptake. However, Syk-independent pathways may also contribute to the TLR4-dependent antigen uptake. Altogether, these murine data suggest an important pro-inflammatory role for mmLDL in macrophage activation through a TLR4-dependent pathway.

Murine TLR4 mRNA is abundant in the lungs, heart, and spleen and, as in humans, is sparse in the muscle, liver, and kidneys (10, 17, 53), although further investigation is needed to clarify the cell types that express it in these tissues.

The TLR4 expression pattern in the mouse CNS differs from the human in that only the microglia expresses TLR4. Neither the mouse astrocytes nor oligodendrocytes do (65). In CNS cell cultures, LPS induces significant injury to developing oligodendrocytes, only in the presence of microglia (76). As oligodendrocytes lack TLR4, these findings suggest that the activation of TLR4 signaling by LPS in microglia leads to indirect injury to oligodendrocyte precursors, which are susceptible to reactive oxygen species and pro-inflammatory cytokines.

Similar to humans, TLR4 is also involved in cardiovascular diseases in mice (67, 77). Murine TLR4 is up-regulated after myocardial infarction and is involved in myocardial dysfunction during bacterial sepsis. TLR4 regulates maladaptive left ventricular remodeling that occur post-infarction, probably via inflammatory cytokine production and matrix degradation. Human and murine pancreatic β-cells express TLR4 as well as CD14 (62). Exposing β-cells to LPS causes the expression of these two molecules to increase in a dose-dependent manner. In the presence of LPS-induced TLR4 expression, in β-cells from both species, insulin mRNA levels, insulin secretion, and the cell viability all decrease.

When infected with *T. cruzi*, liver TLR4 expression levels were found being lower in the C57BL/6 mice than in the BALB/c mice (78). The low level of TLR4 expression contributes to an imbalanced production of pro- and anti-inflammatory cytokines and might play a role in the fatal hepatic injury observed in the infected C57BL/6 mice.

Multiple mouse TLR4 mRNA isoforms can be detected by northern blot analysis (75). A 144-bp insertion between the second and third exon is produced by an alternative splicing. This novel exon contains an in-frame stop codon at 110 bp. The alternatively spliced mRNA encodes a soluble 20-kDa protein (122 amino acids) known as smTLR4 (soluble mouse TLR4), an antagonist to TLR4. smTLR4 mRNA can be detected in various tissues including the brain, heart, lungs, kidneys, liver, thymus, spleen, and small intestine. Although activation of mouse monocytes with LPS causes little changes in the total level of TLR4 mRNA, LPS strongly increases the level of mRNA for smTLR4. The alternatively spliced variant contains a part of the extracellular domain of TLR4 that interacts with LPS (56, 75). Thus, smTLR4 may compete with TLR4 for LPS binding. smTLR4 may suppress LPS signaling by interacting with CD14 and/or LBP or by inhibiting the TLR4/MD-2 interaction. In addition to the secreted smTLR4, a significant amount of smTLR4 is entrapped in the cell membrane, where it may mediate antagonistic effects as well through interacting with newly synthesized TLR4 or CD14 and thus blocking TLR4 signal transduction. As smTLR4 inhibits LPS-mediated signals and smTLR4 mRNA synthesis is induced by LPS, one can hypothesize that smTLR4 is involved in a negative feedback loop to prevent an excessive LPS response. Interestingly, smTLR4 mRNA synthesis also increases in T cells following TCR engagement. Thus, smTLR4 may also regulate the LPS response in T cells. Nonetheless, a human counterpart of the smTLR4 remains to be confirmed.

Lipopolysaccharide increases TLR4 expression in human macrophages and monocytes. In contrast, TLR4 expression decreases in mouse peritoneal macrophages and neutrophils after LPS challenge and remains unaffected in mouse monocytes (55, 79). Pre-exposure to LPS reduces cellular sensitivity to subsequent LPS challenge. This phenomenon, termed LPS tolerance, is mainly due to the loss of surface TLR4 expression. LPS pretreatment of mouse macrophages suppresses inflammatory cytokine production in a time- and dose-dependent manner and significantly reduces NF-κB DNA-binding activity (79). Schroder et al. reported differences in the gene regulation of human and murine macrophages following LPS stimulation (8). Although TLR4 target genes are more rapidly induced in human macrophages than in mouse macrophages following LPS exposure, several negative feedback regulators of the TLR4 pathway are more rapidly induced and to a greater degree in mouse macrophages. This enhanced negative feedback regulation may further reduce the primary LPS response in mouse macrophages, thereby contributing to the lower endotoxin sensitivity in mice compared to humans.

Another difference between mouse and human TLR4 probably lies in the LPS recognition spectrum of the receptor complex. For example, human TLR4, but not murine TLR4, can discriminate between the hexa- and penta-acylated forms of LPS produced by *Pseudomonas aeruginosa* from the airways of cystic fibrosis patients. This discrimination is mediated by the 82-amino acid hypervariable region of TLR4 (44). In addition, differences in the primary structure of MD-2 across species can also lead to changes in TLR4 binding specificities. Using different chimeric complexes consisting of human and mouse TLR4 and MD-2, the MD-2-mediated TLR4 species-specific ligand recognition was demonstrated for Taxol (80) and lipid IVa (a lipid A analog) (81). Taxol, a chemotherapeutic drug derived from plants, acts as a TLR4 agonist on murine cells but not on human cells. Human cells discriminate lipid IVa from lipid A and only respond to the latter, whereas mouse cells respond similarly to both lipids. Lipid A is the anchor moiety and active component of LPS. Variability in its structure has also been found between bacterial species. For instance, lipid A from *E. coli* stimulates human monocytes, leptospiral lipid A does not. By contrast, the lipid A from *Leptospira* activates murine cells (82). In 2009, Vasl et al. reported additional functional differences between human and murine MD-2, including the ability of human but not murine MD-2 to be secreted and to function as an extracellular endotoxin-binding protein with or without TLR4 (36).

#### Rat

In contrast to human pDCs, which do not express TLR4, TLR4 mRNA transcripts have been detected in all rat DC subsets (CD4+ and CD4− myeloid DCs and plasmacytoid DCs) and monocytes from Sprague-Dawley, Lewis and Brown Norway rats. Monocytes, pDCs, and CD4+ DCs express low levels of TLR4, whereas CD4− DCs express moderate levels of TLR4 (83). LPS increases the survival but not the maturation of pDCs, while only modestly enhancing their stimulatory capacities. CD4+ DCs are poorly responsive to TLR4 ligands, whereas CD4− DCs exhibit increased cell survival and maturation following TLR4 stimulation. Activated CD4− DCs in culture produce high concentrations of IL-12p40, low concentrations of IL-10, very low concentrations of TNF-α and no IL-6.

Normal rat cornea and cardiomyocytes express TLR4 (67, 84). Similar to the behavior of cultured human corneal epithelial cells, exposing the cornea of Wistar rats to *Acanthamoeba* up-regulates the expression of TLR4 and inflammatory cytokines, and activates the transcription of MyD88 and NF-κB genes (84). In humans, TLR4 up-regulation is implicated in cardiovascular diseases. In rats, TLR4 expression is enhanced during hypertension (67). Compared to normotensive Wistar–Kyoto (WKY) rats, spontaneously hypertensive rats (SHR) exhibit an enhanced TLR4 distribution predominantly in cardiomyocytes. The TLR4-positive cardiomyocytes in SHR rats homogenously express TLR4 on the membrane as well as in the cytoplasm. In comparison, TLR4 expression in WKY rat cardiomyocytes is restricted to fewer cells and at a lower intensity on a single cardiomyocyte basis. This enhanced TLR4 level in SHR rats is functionally associated with a higher TLR4-dependent pro-inflammatory activity.

Primary rat brain endothelial cells slightly express the TLR4 mRNA under basal conditions (66). Rat brain expresses TLR4 and its expression pattern evolves during the brain development. The number of TLR4-positive cells is considerably lower in P3 and P5 (preterm human equivalents) rat brains than in P7, P9, or P14 (human early childhood equivalent) brains. Inflammatory events often associate with oxidative stress, which in turn affects TLR expression. As in human, oxidative stress induces TLR4 mRNA synthesis in rat cerebral endothelial cells.

Rat microglia clearly expresses TLR4 and CD14. Similar to mice but in contrast to humans, neither astrocyte nor oligodendrocyte precursors express TLR4 in rats. Thus, microglia appears to be the major cell type in the rat CNS able to transduce LPS signals (76, 85). LPS-treated rat microglia from the forebrain induces oligodendrocyte death. LPS does not affect the TLR4 expression in cultured glial cells but LPS-activated microglia produce a wide variety of reactive oxygen species and cytokines. Similar to the mouse, this could explain the microglia-mediated injury to oligodendroglial precursors following LPS exposure since rat oligodendroglial precursors are vulnerable to such mediators.

#### Rabbit

Different from human tissues, in which the highest levels of TLR4 expression are found in the spleen, the highest levels of TLR4 expression in rabbit tissues occur in the lungs and bone marrow. TLR4 is moderately expressed in the kidneys, spleen, heart, and liver and low levels are found in the thymus. TLR4 is constitutively expressed in the normal basilar arteries of rabbits (47, 86).

TLR4 is up-regulated and activated in the basilar arterial wall during cerebral vasospasm after experimental subarachnoid hemorrhage in rabbits, suggesting a role for TLR4-associated signaling pathway in the pathogenesis and development of inflammation in cerebral vasospasm (86). In the normal lungs of rabbits, alveolar macrophages and endothelial cells, but not epithelial cells, were shown to express TLR4 using immunochemistry analysis (47). Granulocytes also expressed TLR4 but to a lesser extent when compared to macrophages, epithelial cells, and endothelial cells. Further studies using the more sensitive flow cytometry technology indicated that TLR4 was located on the surface as well as in the cytoplasm of alveolar macrophages and pulmonary epithelial cells. It is plausible that a cytoplasmic pool of TLR4 allows rapid transport of these receptors to the cell surface. TLR4 translocation and an increased TLR4 gene transcription are therefore two likely mechanisms responsible for the elevated TLR4 expression on the surfaces of these cells observed in inflamed lungs or in the lungs of rabbits suffering from Gram-negative pneumonia (47). The TLR4/MD-2 levels in these infected animals also increase in the bone marrow and kidneys and TLR4 expression is significantly up-regulated in the spleen. Thus, TLR4 in the rabbit likely plays a critical role in the innate response to bacterial infection in the lungs and likely is involved in preventing systemic dissemination of the infection. Mechanical ventilation increases the inflammatory response to LPS via a CD14-independent, TLR4-dependent pathway, as demonstrated using an anti-TLR4 monoclonal antibody (87).

#### Swine

Porcine monocytes, immature and mature mo-DCs express TLR4 transcripts, whereas human mature mo-DCs do not (52). In porcine blood mononuclear cells, LPS induces or up-regulates the transcription of TLR4, CD14, and MD-2 mRNAs, as well as cytokine mRNAs such as IL-1, IL-10, IL-12, TNF-α, and IFN-γ. Synthesis of IL-12p40, IL-6, IL-1β, IL-8, IL-10, and TNF-α proteins also increases (88–90). A transcriptomic analysis of porcine PBMCs stimulated *in vitro* with LPS indicated that most of the up-regulated genes were involved in inflammation and the innate immunity, including chemotactic factors targeting a broad spectrum of innate immune cells (91). Serum amyloid A, which is involved in the establishment and maintenance of inflammation, was the most prominently up-regulated gene. In contrast, LPS exposure induced a strong down-regulation of classical and non-classical MHC-II genes. Although TLR4 expression increases in human monocytes following LPS exposure, the same effect does not occur in porcine monocytes (88).

Among organs, the highest levels of porcine TLR4 mRNA are detected by PCR in the colon and spleen. However, TLR4 mRNA is also found in the lungs, small intestine, liver, kidneys, thymus, lymph nodes, brain (hypothalamus, hippocampus, cortex, and cerebellum), tonsils, ovary, and cornea. As in humans, TLR4 mRNA is undetectable in porcine skin and skeletal muscle (42, 52, 92, 93). Immunohistochemistry studies have revealed that TLR4 in the lungs is expressed by macrophages and epithelium, in the liver by Kupffer cells and by bile duct epithelium. However, neither hepatocytes nor hepatic vascular epithelium express it (93). Porcine neonatal intestinal epitheliocytes (PIE) consistently produce TLR4 and MD-2 mRNAs with a high level of TLR4 protein detected. LPS exposure of PIEs enhances the expression of TLR4, pro-inflammatory cytokines, and chemokines (94). TLR4 is also expressed in M cells and in gut-associated lymphoid tissues. In pigs infected with porcine reproductive and respiratory syndrome virus, TLR4 is up-regulated in tracheobronchial lymph node, brain, hypothalamus, hippocampus and, to a lesser extent, in the cortex and cerebellum. But it is not up-regulated in the lungs (95).

#### Dog

As in humans, the peripheral blood leukocytes of dogs express high levels of TLR4 mRNA. Moderate levels are found in the canine spleen, stomach, small intestine, lungs, and cornea, while low levels in the liver and kidneys. No TLR4 expression was detected in the skeletal muscle and skin (51, 53, 93). The presence of TLR4 mRNA in canine large intestine is controversial. No mRNA was detected by conventional RT-PCR (53) but a recent Real-Time RT-PCR analysis let Burgener et al. detect TLR4 mRNA in both small and large intestines (96). Among the mesenteric lymph nodes, stomach, colon, duodenum, and ileum, the mesenteric lymph nodes contained the highest amount of TLR4 mRNA and the colon contained the least (96). Functional TLR4 was found constitutively expressed at a low level in canine primary colonic epithelial cells and rapidly up-regulated following TLR4 ligand exposure. Activation of TLR4 signaling in these cells by LPS results in secretion of IL-8 and IL-7 (39, 51, 97).

As shown for swine, immunochemistry studies of canine tissues have revealed that TLR4 in the lungs is expressed by macrophages and epithelium, in the liver by Kupffer cells and bile duct epithelium; but it is not expressed by hepatocytes or by vascular epithelium (93).

Overall, due to limited available information describing TLR4 expression and functionality in the rat, swine, rabbit, and dog, it is difficult to make comprehensive comparisons between these species or to the human.

### Response to LPS across species

As discussed in this review, LPS is the primary natural agonist to TLR4. It is important to note that LPS sensitivity differs considerable at the whole-organism level among species (Table 5). Whereas characteristic symptoms of sepsis occur in humans (98) and chimpanzees (99) following an exposure to only 1–5 ng/kg LPS, in baboons and old world monkey species induction of sepsis requires a dose of 0.1–6 mg/kg LPS (100). Rabbits are as sensitive as humans to LPS since 2–4 ng/kg LPS induces an increase in body temperature defined as the threshold level of sepsis symptoms by the United State Pharmacopeia (101). In rabbits, an endotoxin dose of 1–3 μg/kg by intravenous route results in a cardiovascular hyperdynamic state (102), whereas a higher dose at 5 mg/kg leads to a hypodynamic state, that is, a severe septic shock (103). In contrast, swine appears heterogenic in response to LPS. Administration of 10 μg/kg LPS by intravenous route induces a hyperdynamic state in 70% of the animals but a hypodynamic state or fatal septic shock in 30% of the animals (104). Dogs appear similar to swine in the sensitivity to LPS since the dose required to induce a hyperdynamic state is also around 10 μg/kg by intravenous injection (105). Finally, mice and rats are highly insensitive to LPS. Indeed, mice require an intraperitoneal LPS dose of 0.5 mg/kg to cause body temperature increase (106), while intraperitoneal administration of 8 mg/kg of LPS is lethal (107). Rats show a hyperdynamic state of sepsis following an intravenous exposure to 3 mg/kg of LPS (108). An intraperitoneal dose of 15 mg/kg of LPS leads to 90% mortality in rats within 48h following exposure (109) and an intravenous dose of 40 mg/kg of LPS induces a hypodynamic state with 100% lethality within 24 h (110).

**Table 5:** **Comparison of the LPS dose required to induce physiological changes across species, relative to the LPS dose required in humans**.

	Threshold of physiological changes	Severe sepsis or lethal dose
Humans	1–5 ng/kg (i.v.)	
Chimpanzees	1–5 ng/kg (i.v.)	
Baboons and old world monkeys	0.1–6 mg/kg (i.v.)	
Rabbits	2–4 ng/kg (i.v.)	5 mg/kg (i.v.)
Swine		10 μg/kg (i.v.) (30% of animals)
Mice	0.5 mg/kg (i.p.)	8 mg/kg (i.p.)
Rats		15 mg/kg (i.p.) to 40 mg/kg (i.v.)

The divergence in LPS responsiveness between species may partially be attributed to different cytokine production patterns. For instance, LPS exposure of baboon total blood leukocytes induces a high level of CXCL2 mRNA and low levels of CXCL8 and CCL3 mRNAs (99). In contrast, stimulated human and chimpanzee leukocytes express high levels of CXCL8 and CCL3 and a low level of CXCL2. Compared to human cells, at the mRNA level, chimpanzee and baboon total blood leukocytes produce similar TNF-α and IL-10 but higher IL-1β in response to LPS. The level of IL-6 mRNA produced after LPS stimulation is lower in baboon blood leukocytes than in human and chimpanzee leukocytes.

Moreover, it has been noted that the immune response induced by LPS exposure is regulated in an age-dependent manner in humans and macaques, an old world monkey species. When humans are considered, cytokine synthesis in whole blood cells following LPS exposure has been reported to be generally lower in infants than in adults (111). Cord blood cells stimulated with LPS produced lower amounts of TNF-α, IP-10, IL-12p70, and IFN-γ than those produced by stimulated blood cells from adults. In contrast, stimulated cord blood cells produced higher amounts of IL-6, IL-8, and IL-10 than those produced by adult blood cells and IL-1β synthesis remained relatively constant across cord, infant, and adult blood samples. A TLR9 agonist (e.g., CpG) did not induce the same pattern of cytokine release, arguing for a TLR4-dependent LPS effect. When macaques are considered, the numbers of TNF-α or IL-6 secreting myeloid DCs and of IL-6 secreting monocytes are significantly lower in older macaques than in adult macaques (71). In contrast, the numbers of TNF-α secreting monocytes are significantly higher in aged macaques than in adult animals.

## Concluding Remarks

Vertebrate TLRs are highly conserved in their coding sequences, functions, and signaling pathways across species (112). As expected, NHPs have greater similarity to humans than other animal species regarding TLR4 gene and protein sequences, as well as its regulation, down-stream signaling, and function. Although substantial differences exist even between closely related primate species, NHPs are probably the most relevant animal model for the prediction of human responses stimulated by TLR4 agonists. Nonetheless, ethical and financial considerations limit the use of NHPs for routing pre-clinical vaccine evaluation.

The mouse is the most commonly used animal model for vaccine pre-clinical pharmacology studies. Therefore, it is extremely important to be aware of the inherent limitations pertaining to mouse studies as highlighted in this review and by others (10). One should also take into account the differences in the TLR4 systems of various laboratory mouse strains. Such mouse strains as C3H/HeJ and C57BL/10ScCr may not be appropriate especially if the study is designed to assess the effect of TLR4 agonists, due to their genetic defects in the TLR4 locus or in the regulation of the TLR4 gene.

Ample studies have illustrated in humans and mice the expression patterns and functions of TLR4, thus allowing an extensive comparison between the two species. Unfortunately, available information is much sparse for other animal species such as rats, rabbits, swine, and dogs regarding the properties of TLR4. Existing evidence suggests that rabbits and swine may be closer to humans than mice concerning TLR4 sequences and its function. In this regard, humans are highly sensitive to LPS with physiological changes induced by a dose at nanogram per kilogram; swine and rabbits are moderately sensitive to LPS whose physiological changes can be induced by a dose at the microgram per kilogram range; and mice are highly resistant to LPS with physiological changes induced by a dose at milligrams per kilogram (99, 113).

However, one should take caution not to over-interpret the TLR4 similarities or differences reviewed in this article. It is probable that some disparate features seen are attributed to different experimental settings and materials employed in various studies. It has been reported, for example, LPS of different bacteria do not stimulate the same array of cytokine production in a whole blood assay (114). In future, with the prevalence of new generation “omics” technologies, studies can be performed beyond the assessment of a specific pathway in a particular pre-clinical model. Efforts are needed to research in different species for those common elements induced by a similar TLR4 stimulation, preferable using a well-defined high purity TLR4 agonist. Cross-analyses of human data with results from animal studies using a system biology approach can potentially identify those parts in the animal responses that are relevant for humans, thus defining predictive biomarkers and to further refine the animal models used for non-clinical assessment of TLR4 agonists. Such investigations could greatly enhance our ability to translate non-clinical results into accurate predictions of clinical outcomes, in terms of both product efficacy and potential toxicity.

## Conflict of Interest Statement

The authors declare that the research was conducted in the absence of any commercial or financial relationships that could be construed as a potential conflict of interest.
